# Earth’s magnetic field and its relationship to the origin of life, evolution and planetary habitability

**DOI:** 10.1093/nsr/nwaf082

**Published:** 2025-03-03

**Authors:** John A Tarduno, Tinghong Zhou, Wentao Huang, Jaganmoy Jodder

**Affiliations:** Department of Earth & Environmental Sciences, University of Rochester, Rochester, NY 14618, USA; Department of Physics & Astronomy, University of Rochester, Rochester, NY 14618, USA; Laboratory for Laser Energetics, University of Rochester, Rochester, NY 14623, USA; Department of Earth & Environmental Sciences, University of Rochester, Rochester, NY 14618, USA; State Key Laboratory of Tibetan Plateau Earth System, Environment and Resources (TPESER), Institute of Tibetan Plateau Research, Chinese Academy of Sciences, Beijing 100101, China; Centre for Planetary Habitability, Department of Geosciences, University of Oslo, Oslo 0316, Norway; Evolutionary Studies Institute, University of the Witwatersrand, Wits 2050, South Africa

**Keywords:** geomagnetic field, magnetosphere, habitability, origin of life, Ediacaran animal evolution

## Abstract

Earth’s magnetic field history can provide insight into why life was able to originate and evolve on our planet, and how habitability has been maintained. The magnetism of minute magnetic inclusions in zircons indicates that the geomagnetic field is at least 4.2 billion years old, corresponding with genetic estimates for the age of the last universal common ancestor. The early establishment of the field would have provided shielding from solar and cosmic radiation, fostering environments for life to develop. The field was also likely important for preserving Earth’s water, essential for life as we know it. Between 3.9 and ca. 3.4 billion years ago, zircon magnetism suggests latitudinal stasis of different ancestral terrains, and stagnant lid tectonics. These data also indicate that the solid Earth was stable with respect to the spin axis, consistent with the absence of plate tectonic driving forces. Moreover, these data point to the existence of low-latitude continental nuclei with equable climate locales that could have supported early life. Near the end of the Precambrian (0.591 to 0.565 billion years ago), the dynamo nearly collapsed, but growth of the inner core during earliest Cambrian times renewed the magnetic field and shielding, helping to prevent drying of the planet. Before this renewal, the ultra-weak magnetic shielding may have had an unexpected effect on evolution. The extremely weak field could have allowed enhanced hydrogen escape to space, leading to increased oxygenation of the atmosphere and oceans. In this way, Earth’s magnetic field may have assisted the radiation of the macroscopic and mobile animals of the Ediacara fauna. Whether the Ediacara fauna are genetically related to modern life is a matter of debate, but if so, magnetospheric control on atmospheric composition may have led to an acceleration in evolution that ultimately resulted in the emergence of intelligent life.

## INTRODUCTION

When considering how Earth’s magnetic field might be related to the origin of life, evolution and habitability [1–3], it is useful to recall early thoughts on the environment needed for life’s origins. At arguably the most basic level, these works [4–7] required an atmosphere and water, and therefore processes that prevented these from being completely stripped by early intense solar winds streaming from the young Sun become an additional requirement [3]. Shielding by a magnetic field is one process that might prevent such catastrophic loss [3,8]. In contrast, some 60 years ago Sagan [2] suggested that high-energy particles that might be shielded by Earth’s magnetic field could also be seen as a source of energy needed to construct the building blocks of life. Sagan’s suggestion was an early hint of a duality regarding planetary magnetic fields. Strong magnetic fields might under certain conditions provide shielding, preserving a planetary atmosphere and water necessary for life, whereas a weak field might in other circumstances assist life. With this in mind, knowing the exact magnetic field history constrained by observations, rather than modeled values, gains even more importance in our attempts to determine the field’s influence on life’s origin and evolution. In addition to its role in shielding atmospheres and oceans, the magnetic field can provide information of surface processes, including the presence/absence of plate tectonics and polar wander, the rotation of the entire Earth with respect to the spin axis due to shifts in mass heterogeneities composing the planet [9]. These factors in turn bear on the climatic stability of conducive sites for early life.

**Figure 1.**
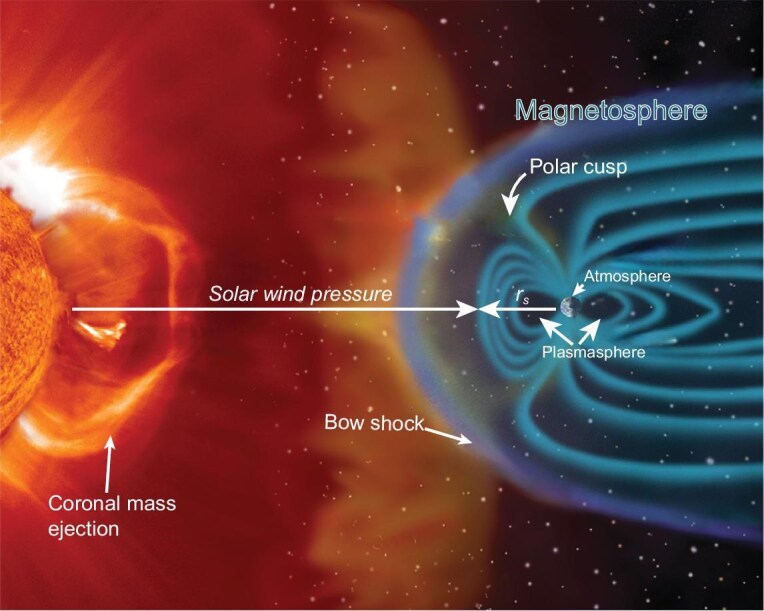
Key features of the solar-terrestrial interaction. The solar wind is a constant stream composed of protons, electrons and alpha particles, and its interaction with Earth’s magnetic field shapes the magnetosphere. The region where solar wind pressure is balanced by the geomagnetic field is the magnetopause. Image credit: ESA/NASA - SOHO/LASCO/EIT.

But magnetic field history provides only one of two crucial inputs needed to gain a first-order understanding of magnetic shielding in deep time. The other required input defining solar-terrestrial interaction is knowledge of past solar winds (Fig. 1). It is convenient to summarize such interaction by the magnetopause standoff distance ($r_{s}$), defined as the point toward the Sun (the sub-solar point) where the solar wind pressure is balanced by the magnetic field [10]:


(1) \begin{eqnarray*}
r_s=\bigg [\frac{\mu _0f_0^2M_E^2}{4\pi ^2(2\mu _0n_{\rm sw}m_{p}v_{\rm sw}^2+B^2_{\rm IMF})}\bigg ]^{1/6}.
\end{eqnarray*}

Here $M_{E}$ is Earth’s dipole moment, $n_{\rm sw}$ is the solar wind density, $v_{\rm sw}$ is the solar wind velocity, $f_{0}$ is a magnetospheric form factor ($= 1.16$ for Earth), $\mu _{0}$ is the permeability of free space, $m_{p}$ is a proton mass and $B_{\rm IMF}$ is the interplanetary magnetic field. The present-day standoff distance is between 10 and 11 Earth radii ($r_{\oplus }$), but decreases dramatically during coronal mass ejections (CMEs)—explosive expulsion events of solar plasma and embedded magnetic field. The standoff distance can be reduced to half of its typical distance on hour timescales. Low-latitude auroras caused by CMEs that induce geomagnetic storms attest to variations in the magnetopause standoff and the magnetosphere in recent times.

Solar wind pressure on timescales of many billions of years relevant for the origin and evolution of life and planetary habitability can be estimated from solar evolution models [3,11], including those based on solar analogs [12]. Paleomagnetic data provide estimates of Earth’s past dipole moment. Solar wind intensities are thought to have been tens to hundreds of times more intense in the Paleoarchean and older times, and this heightens the potential alluded to above of erosion of the atmosphere and water removal in the absence of shielding provided by the geomagnetic field [3].

Defining Earth’s magnetic field in Archean and older times is challenging, especially regarding the dipole moment. Such data must adhere to magnetic recording requirements, including evidence that the magnetization is held by single-domain-like magnetic mineral recorders that preserve primary signals that have not been reset by younger geologic events (see the section entitled ‘Magnetic recording on billion-year timescales’), and that the data average sufficient time to reflect the mean state of the dynamo (see the discussion in the section entitled ‘Evidence for stagnant lid tectonics’). As discussed in further detail below, the single-crystal paleointensity (SCP) method [13,14] addresses these requirements, and has been used to define magnetic field strength progressively further back in time. Specifically, analyses by the University of Rochester Paleomagnetism group led to dipole moment estimates for ca. 2.45 [15], 3.2 [16] and 3.45 billion years ago (Ga) [11]. These estimates were in turn used to estimate steady-state magnetopause standoff distances, which showed a progressive decrease with increasing age from $\sim\! 8r_{\oplus }$ at 2.45 Ga to $\sim\! 5r_{\oplus }$ at 3.45 Ga.

The available SCP dipole moment data, solar wind models and standoffs were summarized in a 2014 review [3]. Herein, we review new findings on Earth’s Precambrian magnetic field from the subsequent decade, focusing on efforts to retrieve the highest-fidelity records. Seminal advances include evidence for a geodynamo as old as 4.2 Ga [17] and the discovery of ultra-low field strengths at 565 Ma [18]. Guided by these major developments, we divide the review into two time intervals not covered previously: the Hadean Eon to Paleoarchean Era, and the Ediacaran Period, and discuss ways in which the field could be related to the origin of life, evolution and habitability.

Because the accuracy of the reconstructed history of the dynamo relies on the foundations of robust field and laboratory work, this review by necessity discusses technical details of the paleointensity analysis and tests of the primacy of magnetic signals. Readers interested in a synoptic view of the implications for the origin of life and evolution are directed to the sections entitled ‘Implications for the origin of life and habitability’, ‘Ultra-low-field-aided oxygenation and Ediacaran evolution’ and ‘Summary, discussion and outlook” that follow below. For a review of the relationship between magnetic fields, extant organisms and Phanerozoic life in general, we refer readers to the review by Pan and Li [19] and the references therein.

## THE HADEAN TO PALEOARCHEAN EONS

Although there may still be outcrops with original primeval paleomagnetic information yet to be discovered, ubiquitous amphibolite and/or higher metamorphism and deformation have erased primary magnetizations from known extant rocks older than $\sim\! 3.5$ Ga. However, an alternative way of retrieving the multi-hundred-million year time sequence stretching into the Hadean needed to address the salient issues of habitability is through the analysis of ancient crystals now found in younger sedimentary units [3]. Zircons hosting magnetite inclusions are an obvious choice: they can be dated by Pb-Pb geochronology and prior studies have argued that they preserve primary isotopic signals.

Measurements of the zircon magnetizations rely on SCP measurement techniques [13,14,20–22], which aim to select samples with single-domain-like carriers that can preserve fields for billions of years, avoiding the multidomain grains that are expected to carry secondary magnetizations. The SCP approach has been affirmed by comparisons of single crystals and whole rocks [23], the latter containing multidomain grains, and by studies of laboratory remanences [24], and these techniques have been adopted in other labs [25–27]. The SCP method employs Thellier double-heating procedures [28–30] that remain the gold standard for paleointensity analysis as they best replicate the thermoremanent magnetization process and are further supported by theory of single-domain magnetic grains established by Néel [31,32].

### Magnetic recording on billion-year timescales

Single-domain grains are minute. For magnetite (Fe$_{3}$O$_{4}$), a typical size is ${\sim\! 50}$ nm for equant grains, but can be larger for elongated and/or needle-shaped particles. If grains are smaller, they are superparamagnetic and they will not have stable magnetizations at room temperature. Importantly, transmission electron microscope or atom probes can be used to image iron nanoparticles, but these are not relevant for paleomagnetism if the grains are in the SP state (see the examples in [33,34], and the discussion of Tarduno *et al.* [35,36]). Hence, nano-paleomagnetism is impossible; meaningful paleomagnetism generally starts at the micro-scale. Moreover, a single magnetic particle, even if in the single-domain state, cannot define a paleomagnetic field, because such definition relies on an assemblage of particles large enough to recover the slight alignment with the ambient field represented by a thermoremanent magnetization. Formally, this distribution requirement is set by Maxwell–Boltzmann statistical limits [37].

If magnetic grains are larger than single-domain sizes, domain walls will form, signifying the multidomain state, which, as described below and revisited in this work, represents a major tripping point for correctly recovering the history of Earth’s magnetic field. Using theory [31,32], we can define the magnetic relaxation time of a single-domain grain as [30]


(2) \begin{eqnarray*}
\frac{1}{\tau }=\frac{1}{\tau _{0}}{\rm {exp}}\bigg [-\frac{\mu _{0}VM_{s}H_{K}}{2kT} \bigg (1-\frac{|H_{0}|}{H_{K}}\bigg )^{2}\bigg ],
\end{eqnarray*}

where $\tau _{0}$ (approximately $10^{-9}$ s) is the time between thermal excitations, $\mu _{0}$ is the permeability of free space, *V* is the grain volume, $M_{s}$ is the spontaneous magnetization, $H_{K}$ is the microscopic coercive force, *k* is Boltzmann’s constant, *T* is temperature and $H_{0}$ is the applied field. In the context of Earth’s early magnetic field, we are interested in the relationship between relaxation time and unblocking temperatures measured in the lab. We can use equation (2) to write a relationship between relaxation times $\tau _{A}$ and $\tau _{B}$ representing temperatures $T_{A}$ and $T_{B}$ [30,38]:


(3) \begin{eqnarray*}
\frac{T_{A} \rm {ln} (\tau _{A}/\tau _{0})}{M_{s}(T_{A})H_{K}(T_{A})} = \frac{T_{B} \rm {ln} (\tau _{B}/\tau _{0})}{M_{s}(T_{B})H_{K}(T_{B})}.
\end{eqnarray*}

This relationship indicates relatively severe constraints on the fidelity of magnetic data from Earth’s oldest rocks. For example, if rocks are heated to low-grade metamorphic conditions for 1 million years, a typical orogenic timescale, even single-domain magnetic grains with blocking temperatures $<\! 400^{\circ }$C are expected to be reset. In the case of multidomain grains common in bulk rock samples, unblocking temperatures will be contaminated up to the Curie temperature of magnetite [30]. Moreover, many of the world’s oldest rocks have seen reheating over even longer intervals, perhaps tens of millions of years. Global magmatic events such as that at ca. 2.7 Ga, and the presence of multidomain grains in rocks such as Eoarchean banded iron formations [39] or Mesoarchean to Paleoarchean basalts [40,41], disqualify these bulk rocks as carriers of primary magnetization [42].

While the magnetism of zircons was measured as early as 1996 at the University of Rochester as part of a survey of potential silicate mineral carriers, the availability of the ultra-sensitive WS Goree direct-current superconducting quantum interference device (dc SQUID) magnetometer [11] allowing three-component measurements of zircons, together with a field study indicating that the Jack Hills (JH) rocks—host to the oldest known terrestrial zircons—might retain primary magnetizations [43], motivated pursuit of the magnetic record of zircons in earnest, which is described below.

### Definition of a 4.2 Ga magnetic field from Jack Hills zircons

More than 12% of detrital zircons from the JH Discovery Site are Hadean in age, and therefore it is a prime target for SCP analyses. Using the WS Goree ultra-sensitive magnetometer, Tarduno *et al.* [17] found that zircons from this site carried high unblocking temperatures that indicated end-member magnetite as the magnetic carrier. An independent assessment found that these data met the Maxwell–Boltzmann statistical limit threshold for field recording [37]. A prerequisite for paleomagnetic studies of ancient detrital zircons is a test to determine whether the rock sampled has maintained a magnetization older than the host rock age. Here we call this a ‘primary magnetization (PM) type-1’ test. To meet this challenge, a zircon microconglomerate test was devised by Tarduno *et al.* [17], whereas individual oriented zircons take the place of sedimentary clasts in the classic conglomerate test [44]. This test demonstrated that the magnetizations of JH zircons from the Discovery Site predated incorporation into the metaconglomerate at ca. 3 Ga.

A difference between zircon SCP and other silicate types is metamictization—expansion of the zircon lattice caused by U decay. This disruption can compromise the crystal internal integrity and provide pathways for late-stage fluids capable of alteration and transport of iron oxides (although not necessarily high-unblocking-temperature phases like magnetite; very low-unblocking-temperature oxyhydroxides are most common [17]). Thereafter, only a small percentage of ancient zircons are expected to have survived this alteration. Tarduno *et al.* [17] separated JH zircons using non-magnetic techniques by hand and applied selection criteria to exclude zircons compromised by metamictization. However, because detrital minerals could have seen a thermal history after crystallization, additional tests are needed to search for this alteration. Tarduno *et al.* [17] used SHRIMP U-Pb data to test for this thermal signal. Evidence of discordance was searched for in the data that might indicate resetting, and data were examined for non-systematic variations in Pb during individual analyses, seen in SHRIMP analyses of other reheated zircons. We call these ‘PM type-2’ tests.

**Figure 2.**
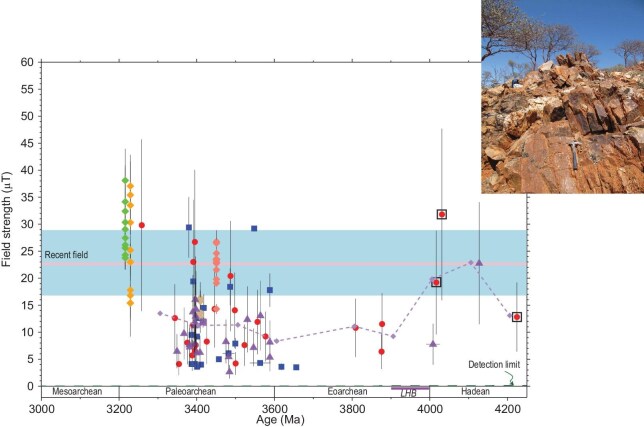
Hadean to Paleoarchean paleointensity history from zircon magnetism of the Jack Hills, Western Australia. Zircon paleointensity: blue triangles, full Thellier determinations; red circles, $565^{\circ }$C Thellier estimates from Tarduno *et al.* [17]; purple triangles, $565^{\circ }$C Thellier estimates from Tarduno *et al.* [35] together with single-crystal paleointensity results from extant rocks (large diamonds). Paleointensity uncertainties shown are 1$\sigma$. Boxes are data with $^{7}$Li constraints, indicating that the samples have not been reheated since formation to reset the magnetization of their included magnetite particles. Dashed line with small diamonds shows the sliding window average (see Tarduno *et al.* [35]). Detection limit represents the hypothetical induced magnetic field strength in the absence of a core dynamo. Pink line is the recent field referenced to the equator with 800 kyr variation (blue). Inset shows the Jack Hills Discovery Site (Western Australia) where zircon host rock samples were collected. Photo from J. Tarduno. Graph from Tarduno *et al.* [35], licensed under CC BY-NC-ND 4.0.

A painstaking screening process led to the rejection of over $\sim\! 98\%$ of thousands of crystals separated from the JH Discovery Site. The remaining select data—the first published paleointensity analyses of zircons—defined a geodynamo as old as 4.2 Ga [17] (Fig. 2), extending the geodynamo record by $\sim\! 750$ million years, and indicated that planetary magnetic shielding was in place during the Hadean Eon.

### Replication of the Jack Hills zircon paleointensity data and refined fidelity tests

**Figure 3.**
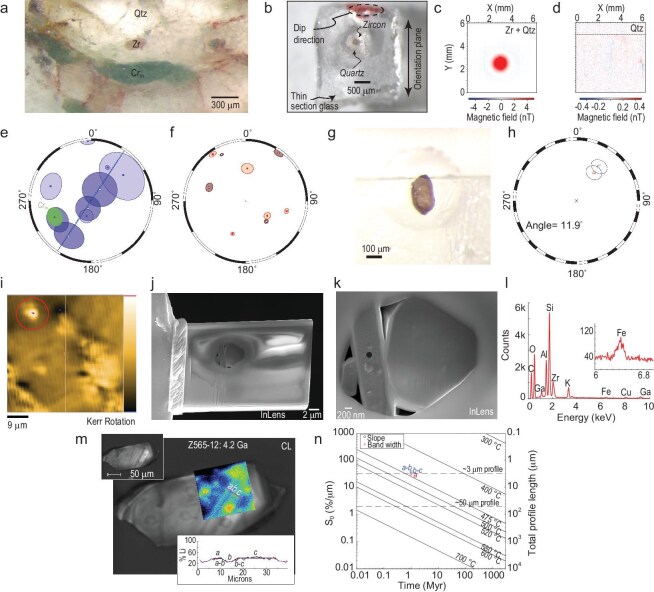
Jack Hills zircon magnetism tests demonstrating primary magnetic signals. (a) Photomicrograph of a sample from the Jack Hills with zircon (Zr), the chrome mica (Cr$_{\rm {m}}$) fuchsite and quartz (Qtz) highlighted. (b) Illustration of the technique to obtain an oriented zircon from the Jack Hills metaconglomerate host rock. Each oriented zircon has some surrounding quartz. Subsequent measurement of only the zircon (c) with a scanning SQUID magnetometer (AIST, Japan) shows a clear signal, whereas the surrounding quartz (d) has no signal, demonstrating that oriented zircons can be used for a microconglomerate test. (e,f) Microconglomerate test results. The magnetizations isolated at temperatures less than the peak metamorphism (e) are not random, but follow a great circle path close to the direction obtained from the fuchsite, interpreted to represent the field during peak heating at 2.65 Ga [46]. The recording of this expected secondary magnetization represents a positive inverse microconglomerate test [46]. Magnetizations isolated at higher unblocking temperatures (f) cannot be distinguished from a random distribution and represent a positive microconglomerate test, and a positive type-1 primary magnetization test described here. (g) Jack Hills zircon natural remanent magnetizations have been reproduced by measurements of the same zircon at the University of Rochester using the WSG ultra-sensitive SQUID magnetometer and the scanning SQUID microscope at AIST Japan [35]. (i) NanoMOKE3 image of the surface of a Jack Hills zircon showing the magnetic signal highlighted by a red circle. (j) Focused ion-beam slice through the magnetic signal of (i), revealing a buried melt inclusion that is composed of distinct crystals (k) (scanning electron backscatter image), including feldspars and quartz. (l) Electron dispersive spectroscopy reveals iron signals in the feldspars. Images and data from Tarduno *et al.* [35]. (m) Scanning electron microscope backscatter image of a 4.2 Ga Jack Hills zircon, with secondary-ion mass spectrometry (SIMS) $^{7}$Li map (data collected with a Cameca IMS 7f by M. Fayek, University of Manitoba) overlay (blue-red colors). The $^{7}$Li bands in the zircon (cf. the cathodoluminescence (CL), upper right inset) are preserved, and their thicknesses measured (lower right inset). (n) Comparison of $^{7}$Li bands of (m) using the method of Trail *et al.* [49] to estimate limits of reheating. These data indicate that reheating has been limited to temperatures associated with peak metamorphism that has affected the Jack Hills ($475^{\circ }$C) since the 4.2 Ga crystallization of the zircon. Panels from Tarduno *et al.* [35], licensed under CC BY-NC-ND 4.0.

A follow-up study of the JH zircon Discovery Site was conducted by Tarduno *et al.* [35] to determine if the data could be replicated and to expand the tests for primary remanences (Fig. 3). At the same time, that study systematically addressed claims by a competing group trying to disprove prior zircon magnetization studies, and showed how these claims violated fundamentals of magnetic recording and were therefore spurious (see also the Supplementary Information in [35]). In the follow-up study, a low-unblocking-temperature magnetic overprint on the JH zircons was defined for the first time (Fig. 3a–f). Such an overprint is expected because the metaconglomerate hosting the zircons had seen greenschist grade heating to $\sim\! 475^{\circ }$C at 2.65 Ga [45]. The overprint isolated agreed with the direction carried by metamorphic fuchsite carrying relict Fe-Cr grains (Fig. 3a), documented in a study by Cottrell *et al.* [46]. The fuchsite positive ‘inverse’ conglomerate test direction [46]—obtained from the same JH Discovery Site sample studied by Tarduno *et al.* [17]—was interpreted to represent the ca. 2.65 Ga magnetic field direction. The zircon low-unblocking-temperature overprint also agreed with the overprint defined by cluster analyses of paleomagnetic data from JH cobble size clasts found about 1 km from the JH Discovery Site studied by Bono *et al.* [47,48], which had also been interpreted to represent the ca. 2.65 Ga magnetic field direction. The difference in zircon high-unblocking-temperature magnetization directions and this consistent overprint confirms that the JH zircons preserve signals predating deposition of the conglomerate, forming a robust positive PM type-1 test (Fig. 3e–f).

**Figure 4.**
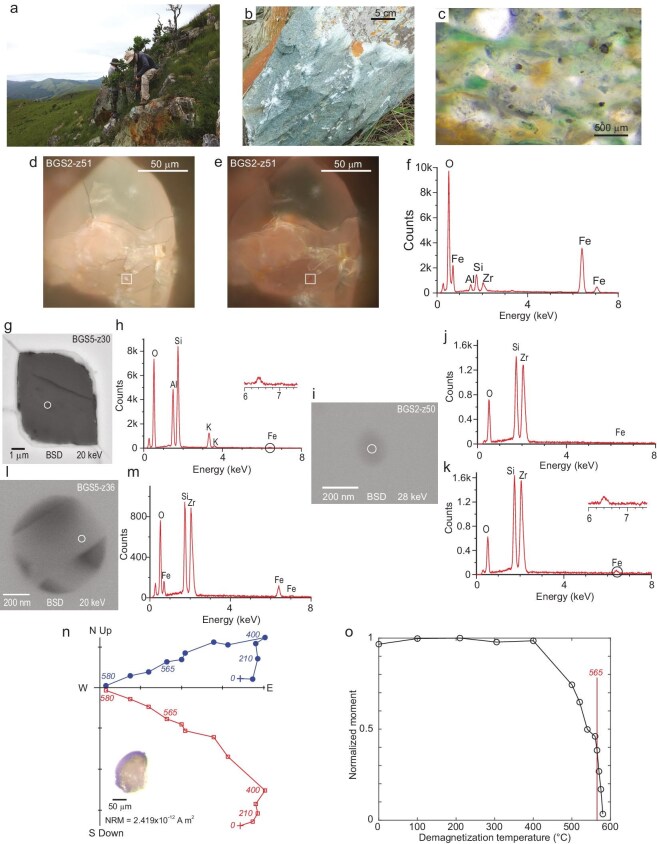
Field setting thin section and scanning electron microscope images of zircons and their magnetite inclusions. (a) Sampling Green Sandstone, Barberton Mountains, South Africa (photo from J. Tarduno). (b) Green Sandstone Bed (photo from J. Tarduno). (c) Thin section of Green Sandstone showing abundant zircons. (d) Reflected light microscope image (100$\times$) of zircon with the magnetite inclusion highlighted (box). (e) Image showing extinction of the magnetite grain ($90^{\circ }$ polarization). (f) Energy dispersive spectroscopy (EDS) of magnetite inclusion highlighted in (d–e). (g) Feldspar inclusion with the EDS analysis spot highlighted that shows Fe (h) and the potential for magnetic inclusions. (i) Buried inclusion with the EDS analysis spot highlighted (circle). (j) EDS analysis of (i) at 20 keV without a clear Fe signature. (k) EDS analysis of (i) at 28 keV; the greater depth penetration showing a Fe signature demonstrates that the Fe oxide inclusion is at depth ($>4\, \mu$m). (l) Melt inclusion with the EDS analysis spot highlighted (circle). (m) EDS analysis shows that crystals within the melt inclusion are iron oxide and potential remanence carriers. (n) Orthogonal vector plot of thermal demagnetization of zircon shown as inset. Temperatures shown are in degrees Celsius. (o) Normalized remanent moment versus thermal demagnetization temperature of Barberton Green Sandstone zircon, with $565^{\circ }$C temperature highlighted. Near complete demagnetization by $580^{\circ }$C indicates a magnetite carrier, whereas the orthogonal vector plot (n) and thermal decay characteristics support use of $565^{\circ }$C as an ideal temperature for the Thellier estimate of the field strength. Panels (b) and (d–o) from Tarduno *et al.* [36], licensed under CC BY 4.0.

A multifaceted approach was used to supplement the PM type-2 tests (Fig. 3g–h). Through reflected light microscopy, scanning electron microscopy (SEM), energy dispersive spectroscopy (EDS) and microstructural analyses, this study also showed that primary magnetite inclusions are present in the interiors of JH zircons (see Fig. 3 (p. 2312) and Fig. 4 (p. 2313) of Tarduno *et al.* [35]), as predicted from the unblocking temperature data in the original study of JH zircons [17], and within the single-domain-like recording size range. Magnetic signals at depth within JH zircons were documented using the magneto-optical Kerr effect, and linked to iron-bearing silicates within multi-component melt inclusions using focused ion-beam sections and subsequent SEM and EDS analyses (see Figs 2–4 of Tarduno *et al.* (p. 2314] and Fig. 3i–k).

Tarduno *et al.* [35] also used $^{7}$Li profiling on zircons reported in the original study of JH zircons [17] to further test reheating prior to zircon deposition (Fig. 3m–n). The Hadean data best represented by a 4.2 Ga zircon, were found to preserve narrow $^{7}$Li bands. Following the calibration of Trail *et al.* [49] this indicates less than $475^{\circ }$C of reheating. Amongst the final 45 selected zircons in the Tarduno *et al.* [17] study one was suspected of thermal exposure prior to incorporation into the metaconglomerate on the basis of Pb-Pb data (cf. sample Z565-5 of Tarduno *et al.* (p. 524] and the discussion above). The $^{7}$Li profiling approach yielded a higher temperature ($550^{\circ }$C; see Fig. S9 and Table S3 of Tarduno *et al.* [17,35]) for this zircon. The preservation of these differences suggests that the $^{7}$Li technique is accurately gauging past temperatures (cf. Tang *et al.* [50] versus Trail *et al.* [49]) for the JH zircon compositions.

Tarduno *et al.* [35] also further demonstrated the resolving power of their Pb-Pb screening techniques by showing how a zircon with a 4.3 Ga core exhibited non-systematic Pb change versus depth—a signal that had been claimed to be unmeasurable by critics—leading to its rejection as a primary magnetic recorder. Consistent with the Pb depth variation this zircon showed a range of ages derived from different analysis spots hence proving critics wrong.

Tarduno *et al.* [35] generated a new zircon paleointensity dataset and these values replicated prior analyses defining a pattern of high late Hadean field strength (Fig. 2). This supports a primary remanence (i.e. a positive PM type-2 test) because the pattern should not arise if the magnetizations are secondary. This history was proposed to record chemical evolution of the core including potential early chemical precipitation that might help power the dynamo (see Tarduno *et al.* [35, p. 2317]).

### Reproducibility of the Jack Hills zircon paleointensity history using Barberton Green Sandstone zircons

If the JH zircons are recording the Paleoarchean to Hadean geomagnetic field, zircons from other localities should record similar signals. Discovery of Hadean zircons in the Green Sandstone Bed (BGS) of the Barberton Mountains of South Africa [35,51] provided the opportunity for such a test (Fig. 4a–b). Zircons are relatively abundant in the bed (Fig. 4c). Upon isolation thermal demagnetization reveals a high-unblocking-temperature magnetization and near complete removal of the natural remanent magnetization after treatment at $580^{\circ }$C, the Curie temperature of magnetite [36] (Fig. 4n–o). The Barberton Mountains have seen lower-grade peak temperatures [52] than the JH Discovery Site and prior conglomerate tests attest to the preservations of high-unblocking-temperature magnetizations [53,54].

In the first study of JH zircons Tarduno *et al.* [17, p. 4] inferred that ‘Sources of magnetite within the JH zircons include isolated inclusions trapped in the zircon matrix vapor phase magnetite associated with voids, and magnetite inclusions within other silicates trapped in the zircons’. This inference proved to be prescient as evidenced by the reflected light, SEM and EDS analyses presented by Tarduno *et al.* [17,35]. These occurrences also apply to the BGS. Specifically Tarduno *et al.* [36] conducted extensive reflected light SEM and EDS analyses and documented the presence of isolated magnetite inclusions in the single-domain and/or single-vortex size range in the BGS zircons, Fe inclusions within voids that are possible vapor phase particles, as well as Fe inclusions with other melt inclusions (Fig. 4d–m). All of these occurrences were found sporadically within the zircon volume such that one cut might reveal little or no inclusions, whereas subsequent polishing to examine deeper levels in the zircon crystal often revealed many inclusions.

**Figure 5.**
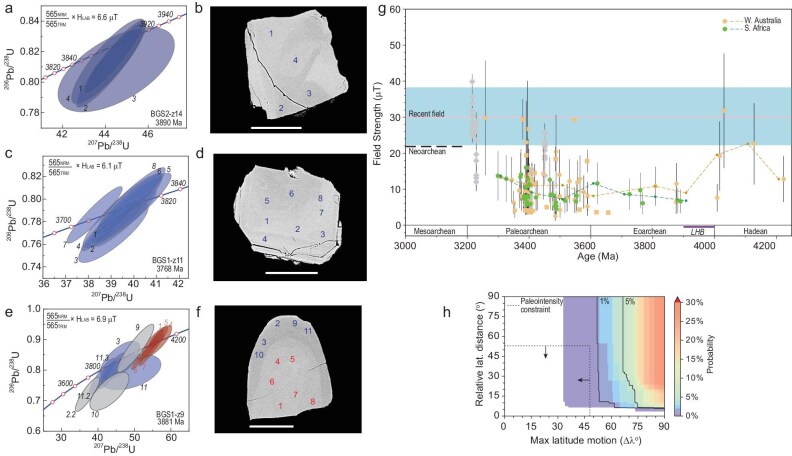
Geochronology paleointensity and implications for surface tectonics derived from Barberton Green Sandstone zircons. (a) U-Pb SHRIMP geochronology (analyses of W. Davis and N. Rayner GSC) for the zircon shown in backscatter scanning electron microscope image (b) with analysis locations numbered, yielding an Eoarchean age. (c) U-Pb SHRIMP geochronology for the zircon shown in backscatter scanning electron microscope image (d) with analysis locations numbered, yielding an Eoarchean age. (e) U-Pb SHRIMP geochronology for the zircon shown in backscatter scanning electron microscope image (f) with analysis locations numbered. The core yields a Hadean age, whereas the thick rim yields an Eoarchean age, suggesting that this zircon can retain magnetization only of Eoarchean age. (g) Paleointensity versus time comparing data from the Barberton Green Sandstone (green) with data from the Jack Hills (yellow). Here the recent field is referenced to the paleolatitude of Mesoarchean data [16]. The near constant values between 3.9 and ca. 3.4 Ga suggest latitudinal stasis. (h) The probability of two ‘plates’ having the relative latitude and absolute latitude motion characteristics shown in (g) based on plate tectonics of the last 600 million years (dashed lines and arrows) is less than 1%. Figure panels from Tarduno *et al.* [36], licensed under CC BY 4.0.

After paleointensity experiments, the next step in BGS analyses involved SQUID U-Pb age determinations (Fig. 5). In these data, a striking difference was seen for the oldest (Hadean) zircons relative to those of the JH Discovery Site: the former showed evidence for an Eoarchean or younger thermal event. This is manifested by Hadean-age cores and younger rims (Fig. 5e–f). However, Eoarchean-age zircons were identified and probing with multiple analysis spots showed no evidence of younger ages (Fig. 5a–d). The BGS zircons studied by Tarduno *et al.* [36] are in general better preserved than those of the JH Discovery Site but to isolate zircons meeting all selection criteria the rejection rate is still high (96.5% for BGS).

The 35 selected BGS zircons traced a paleointensity history between 3.2 and 3.9 Ga (Fig. 5g) that can be compared with that from the JH. These records show remarkable agreement (Fig. 5g). Given that the BGS and JH zircons have seen different geologic histories after deposition in their respective sedimentary host rocks this further confirms the fidelity of the zircon record (and represents a more global positive ‘type-2’ test). Moreover the agreement of the paleointensity values indicates that the JH and BGS were formed in similar latitudes between ca. 3.4 and 3.9 Ga. Below we further explore the implications of the combined JH and BGS records.

### Evidence for stagnant lid tectonics

The time-averaged field is needed to understand the evolution of the dynamo and long-term character of the magnetosphere because of the spatially and time-varying fields described by the Gauss coefficients $g_l^m(t)$ and $h_l^m(t)$ in the scalar potential $\Psi _{m}(r \theta , \phi , t)$:


(4) \begin{eqnarray*}
\Psi _{m}(r, \theta , \phi , t) &=& \frac{r_{\oplus }}{\mu _{o}} \sum _{l=1}^{\infty } \sum _{m=0}^{l} \bigg ( \frac{r_{\oplus }}{r} \bigg )^{l+1} P_{l}^m {\rm {cos}}\, \theta\\
&&\times [ g_{l}^{m}(t) {\rm {cos}} \, m \phi\! +\! h_{l}^{m}(t) {\rm {sin}} \, m \phi ]. \\
\end{eqnarray*}

Here the $P_{l}^m$ are partially normalized Schmidt functions, and *l* and *m* are the spherical harmonic degree and order, respectively. More specifically, enough time must be averaged such that the geocentric axial dipole ($g_1^0$) dominates. This is generally taken as requiring uniform sampling averaging over at least many tens of thousands of years. If this averaging is achieved and a paleomagnetic dipole moment (PDM) constrained, the paleomagnetic inclination can be used to define past latitude, according to the well-known dipole equation


(5) \begin{eqnarray*}
\rm {tan} \, I = 2\, \rm {tan}\, \lambda .
\end{eqnarray*}

And if time averaging has been achieved, paleomagnetic strength *B* can be related to the paleolatitude ($\lambda$) by


(6) \begin{eqnarray*}
B = \frac{\mu _{o} \rm {PDM}}{4\pi r^{3}} \sqrt{1 + 3 \, \rm {sin}^{2} \lambda }.
\end{eqnarray*}

This background is important for understanding the zircon paleointensity record. Field-geometry constraints are unavailable before the Neoarchean [55] but recent studies indicate the lack of an inner core for the timespan considered here [18,56–58]. Modeling of the geodynamo without an inner core suggests a dipole-dominated field [59]. The JH and BGS data show some time averaging due to slow cooling of their host magmatic crustal rocks. Variations in paleointensity of time-averaged values over hundreds of millions of years similar to trends defined for the better-known present-day to Mesozoic paleointensity record, are ascribed to either changes in latitude or changes in the core-mantle boundary [21,22,60–62]. Both are taken as signatures of plate tectonics, the latter driven by deep subduction.

The near constant paleointensity over some 500 million years (i.e. 3.9 to ca. 3.4 Ga) indicates a very different regime for Earth. The pattern of core-mantle heat flow appears to not have changed as would otherwise be expected if deep subduction had been operating, and latitudes are not changing. This raises the longstanding question about the tectonic regime of the early Earth [63,64]. Geochemical indicators of subduction have been used to argue for a Hadean start to plate tectonics [65]. But plate tectonics describes a kinematic framework for the surface. So *sensu stricto* a means of measuring distance defining large-scale plate motion is required to ascribe a modern style of plate tectonics to the early Earth, and this is not provided by geochemistry. To assess whether the latitudinal pattern could be consistent with modern plate tectonics, Richard Bono in Tarduno *et al.* [36] surveyed plate motion histories for the continents over the last 600 Myr [66] to trace the characteristic maximum latitudinal travel. The result indicated a very low probability ($<\!\! 1\%$) that the data sample a modern tectonic regime (Fig. 5h). Instead the data are consistent with stagnant lid tectonics [67] for some 500 million years. Implications of these data for life and habitability are reviewed below.

### Implications for the origin of life and habitability

The identification of a relatively robust geodynamo in the Hadean at 4.2 Ga followed by latitudinal stasis and a subdued geodynamo between 3.9 and ca. 3.4 Ga has several profound implications for life and planetary habitability. Implications for core processes will be addressed separately in future work. Processes of magnetic shielding have been debated. Some authors infer relatively minor atmospheric losses unless long timescales are involved [68] whereas others emphasize that when energy and momentum factors are considered, for most of Earth’s history, the field has had a net protective role [69]. The early field could have helped prevent atmospheric blowoff and also helped limit the deep penetration of the most energetic ionizing radiation to the surface. The latter is important when considering environments for the origin of life. Specifically, recent molecular clock analyses assign an age for the last universal common ancestor (LUCA) at $\sim\! 4.2$ Ga [70]. This suggests that an ecosystem existed possibly relying on a H-rich environment [70]. The presence of magnetic shielding could expand somewhat the range of near surface environments where LUCA could have thrived.

The geochemical cycling provided by plate tectonics is sometimes quoted as being essential for life but the zircon magnetic record indicates that life could have arisen and developed throughout the Eoarchean to Paleoarchean [71,72] in a stagnant lid regime. Hence, on the basis of these data we conclude that plate tectonics is not essential for life during the first billion years of a terrestrial-like planet. The lack of deep subduction could have also subdued polar wander [73]. Large and relatively rapid latitudinal shifts of sites would have been detrimental to nascent life and the lack of these changes could have further fostered evolution on the early Earth. Finally we note that because of the latitudinal stasis the Paleoarchean to early Eoarchean paleointensity values can be linked to paleolatitudinal constraints from analyses of extant Paleoarchean rocks [11]. This tie indicates that there were low-latitude continental sites available for the development of life throughout the Eoarchean to Paleoarchean times [36].

## THE EDIACARAN PERIOD

**Figure 6.**
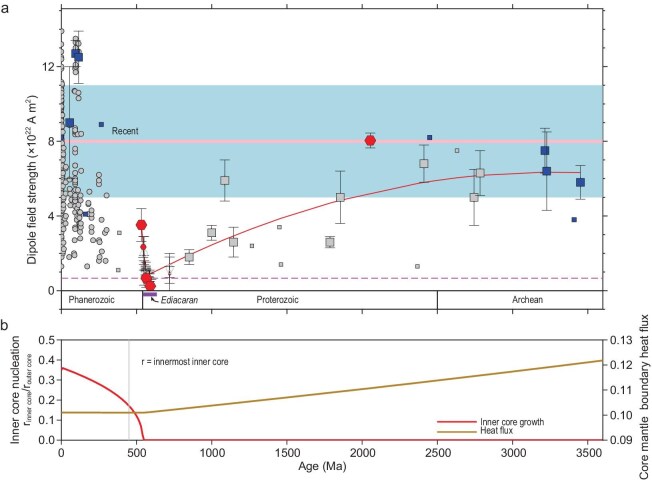
Paleointensity history. (a) Magnetic field strength versus age; updated summary from Bono *et al.* [18], Zhou *et al.* [77], Huang *et al.* [78] and Zhou *et al.* [100]. Filled symbols are Thellier paleointensity results. Blue and red symbols are single-crystal results with the red symbols data since 2019. Large symbols are time-averaged data. Open symbols are Cryogenian-Ediacaran non-thermal data (see Fig. 8). Fit to data (red lines) from 3450 to 565 Ma from Bono *et al.* [18] and fit from 565 to 532 Ma from Zhou *et al.* [77]. Circles are virtual dipole moment data. (b) Inner core radius and core-mantle boundary (CMB) heat flux. Dashed line shows when the radius of the inner core ($r_{\rm ic}$) is 0.5 of that today. Plots from Zhou *et al.* [77], licensed under CC BY 4.0.

Paleomagnetism can shed light on the age of the inner core [18] and in so doing provide the foundational boundary condition for understanding inner core structure [74–77] and potential linkages with changing environmental conditions and the evolution of life [78]. The key signal is an ultra-low field strength before inner core nucleation (ICN) associated with waning thermal energy to power the geodynamo from core-mantle boundary (CMB) heat flux followed by a sharp increase in field intensity as new thermal and compositional sources of buoyancy become available once nucleation commences [77]. The discovery of an ultra-low time-averaged geomagnetic field 10 times weaker than the present day at 565 Ma during the Ediacaran Period by Bono *et al.* [18] provided evidence for the first part of the ICN paleomagnetic signature (Fig. 6a).

This observation together with over a dozen studies indicating unusual field behavior (e.g. [79]) including a hyper-reversal frequency and high dispersion of magnetic directions [80] suggested that the Ediacaran field may have been approaching the weak field state where kinetic energy exceeds magnetic energy. Another observation made by Bono *et al.* [18] was a gradually decreasing field strength over the 1.5 billion years preceding the Ediacaran with superimposed variations that might reflect changes in dynamo efficiency in turn caused by changes in the CMB structure [62]. The long-term signal (red curve in Fig. 6a) could mark the gradual decay of the thermally driven dynamo (e.g. [81]). Based on these observations Bono *et al.* [18] suggested an Ediacaran age of ICN as also predicted in the numerical dynamo modeling of Driscoll [58].

To test the interpretations put forth by Bono *et al.* [18] paleointensity data younger during and older than the Ediacaran have been sought from several global sites. Below we review these data and follow this with a discussion of a hypothesis these results have motivated relating Ediacaran magnetic fields to the radiation of animal life.

### Paleointensity prerequisites to define the mean state of the Ediacaran-Cambrian dynamo

In contrast to the Hadean to Paleoarchean interval extant rocks not heated to amphibolite grade are preserved at numerous sites to study the Ediacaran-Cambrian dynamo. Recalling equations (2)–(4) our criteria for robust data defining the mean state of the past geomagnetic field from these extant rocks include (i) time averaging (ii) Thellier paleointensity analyses and (iii) samples with single-domain-like magnetic particles. Time averaging over many tens of thousands of years is needed to exceed the influence of short-term fluctuations to confidently recover changes in the dipole signal, and hence this represents criterion (i). This can be achieved by sampling many time-independent dikes or lavas and ensuring that paleointensity data represent dispersion of the field [13,14,21,22], or by sampling intrusive rocks that cooled slowly. Bono *et al.* [18] took the latter approach by sampling the slowly cooled anorthosites of the Sept Îles intrusive suite [82] that cooled over at least $\sim\! 75$ kyr. Thus the ultra-low field defined by Bono *et al.* [18] lasted many times longer than the duration of a geomagnetic field reversal.

As described earlier Thellier analyses [28] criterion (ii) remain the gold standard for recovering paleointensity data from samples having thermoremanent magnetizations (TRMs). In addition partial thermoremanent magnetization (pTRM) tests [83] are needed to ensure that samples have not altered during the heatings required by the method. As also highlighted earlier these methods rely on the magnetic mineral recorders being in the single-domain (SD) or single-domain-like (single-vortex or small pseudosingle-domain) state criterion (iii) because only the SD state has a well-established theory [31,32] providing the foundation for accurate paleointensity recording [30]. Most bulk rock samples contain much larger multidomain (MD) magnetic particles tens to hundreds of microns in size and these are a substantial obstacle to uncovering accurate paleointensities. As noted earlier the SCP technique was developed at the University of Rochester to avoid experimentally induced and natural alteration of magnetic mineral carriers and to address the challenge of criterion (iii) [13,14,20–23].

### Cambrian renewal of the dynamo

Zhou *et al.* [77] used the paleointensity criteria noted above and sampled the early Cambrian anorthosites of the Glen Mountains Layered Complex (Wichita Mountains Oklahoma; [84,85]) to help fill a conspicuous gap in the paleointensity history. These rocks, which are well dated with a U-Pb zircon age of $532.49 \pm 0.12$ Ma [86] can average the field by virtue of their relatively slow cooling, estimated by to be $\sim\! 500$ kyr [86]. Using SEM and EDS Zhou *et al.* [77] documented that plagioclase hosted minute titanomagnetite needles similar to those seen in other silicates [87] and in this case typically $\sim\! 200$ nm in width and several micrometers long. Magnetic hysteresis and first-order reversal curve (FORC; [88]) analyses confirmed the SD behavior of the plagioclase crystals. Zhou *et al.* [77] used SCP analyses and after cooling rate and anisotropy corrections reported a value of $13.7 \pm 3.4\,\mu$T and a paleomagnetic dipole moment of $3.5 \pm 0.9 \times 10^{22}$ A m$^{2}$. This time-averaged dipole moment is five times greater than that of the ultra-low Ediacaran Period defined by Bono *et al.* [18]. This relatively rapid renewal of the field is consistent with the new sources of energy to drive the dynamo following ICN. Together with data defining the ultra-low strengths at 565 Ma Zhou *et al.* [77] proposed an ICN age of $\sim\! 550$ Ma (Fig. 6a–b).

Zhou *et al.* [77] also reviewed other whole-rock paleointensity results from Ediacaran to Cryogenian lavas and dikes that had been reported after the Bono *et al.* [18] study. Shcherbakova *et al.* [89] reported Thellier results from Ediacaran lavas and dikes of Ukraine which yield similar ultra-low fields as those reported by Bono *et al.* [18]. The Shcherbakova *et al.* [89] data were subsequently confirmed through more comprehensive sampling and analyses reported by Thallner *et al.* [90]. Other data from the Cambrian Ediacaran and Cryogenian from Canada [91–93] are more difficult to interpret and illustrate the difficulties of paleointensity analyses using whole rocks containing MD grains; only a few Thellier experiments were successful. In contrast Zhou *et al.* [77] reported 17 successful SCP Thellier analyses and Huang *et al.* [78] discussed below reported 43 successful SCP Thellier analyses. Even though they are based on detailed experiments the whole-rock values represent instantaneous records of the field (equation (2)) and are too limited to define the mean state of the dynamo. Nevertheless notwithstanding objections to non-Thellier data [30] these values are largely consistent with the preferred history described by Zhou *et al.* [77] (Fig. 6a). We revisit the use of whole rocks with MD grains below.

### A first-order test of the ultra-low fields using single-crystal paleointensity analyses

**Figure 7.**
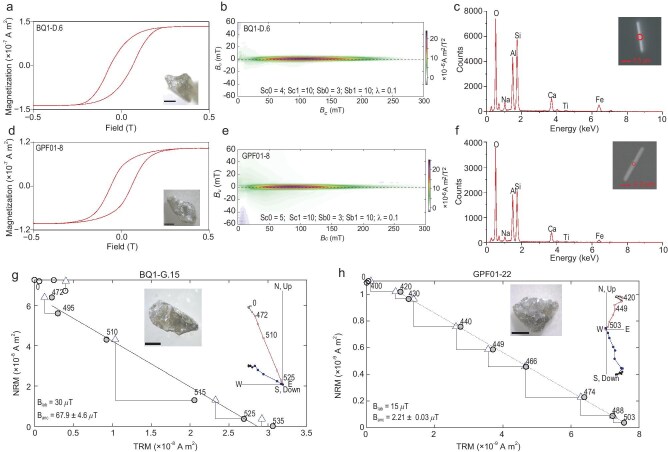
Rock magnetic (magnetic hysteresis data), scanning electron microscopy and paleointensity data for 2054 Ma (Paleoproterozoic) feldspars versus 591 Ma (Ediacaran) feldspars. Data for 2054 Ma crystals are from pyroxenites of the Bushveld Complex and are as follows: (a) magnetic hysteresis loop, (b) first-order reversal curve, (c) energy dispersive spectroscopy with the inset showing the magnetic particle. Data for 591 Ma crystals (d–f) from the Passo da Fabiana gabbro; plots follow the conventions in (a–c). (g) Paleointensity data for the 2054 Ma feldspar; crystal measured is shown in the inset. Natural remanent magnetization (NRM) lost versus thermoremanent magnetization (TRM) gained (circles) with partial thermoremanent magnetization checks shown by triangles. Best fit line shown; gray circles are data used in the fit. Orthogonal vector plot of field off steps shown in the inset; red/blue portions used in the paleointensity fit. Here ${\rm B}_{\rm {lab}}$ is the applied field, ${\rm B}_{\rm {anc}}$ is the calculated ancient value. (h) Paleointensity data for the 591 Ma feldspar following the conventions in (g). Despite nearly identical recording properties, the Ediacaran crystal yields a paleointensity $>\!30$ times weaker than the Paleoproterozoic feldspar. Figure panels from Huang *et al.* [78], licensed under CC BY 4.0.

Huang *et al.* [78] conducted a test of the time-averaged Ediacaran ultra-low fields and the decay of the field from earlier higher values. Huang *et al.* [78] identified two sets of rocks one emplaced in the Ediacaran Period and the other much older having plagioclase feldspar crystals with very similar needle-like titanomagnetite inclusions with SD characteristics defined by magnetic hysteresis [94] FORC measurements [88] and SEM/EDS analyses (Fig. 7).

Feldspar-bearing pyroxenites of the Bushveld Complex of South Africa, well dated by U-Pb zircon geochronology at $2054 \pm 1.3$ Ma [95] composed the set older than the Ediacaran Period. The other unit Passo da Fabiana gabbros of Brazil, which are part of the Dom Feliciano Belt that also extends into Uruguay [96] are well dated with a U-Pb zircon age of $591.2 \pm 3.5$ Ma [97] and are of Ediacaran age. Both units are slowly cooled intrusive rocks; Huang *et al.* [78] estimated that the Bushveld samples cooled through the temperature range holding the remanence ($\sim\! 360^{\circ }$C to $\sim\! 580^{\circ }$C) in $\sim\! 140$ kyr, and that the Passo da Fabiana gabbros cooled on similar timescales.

After cooling rate and anisotropy corrections, the Bushveld SCP analyses yielded a field value of $48.6 \pm 3.0\, \mu$T, and a paleomagnetic dipole moment of $8.04 \pm 0.40 \times 10^{22}$ A m$^{2}$. In contrast, after cooling rate and anisotropy corrections, SCP data from the Passo da Fabiana gabbros yielded a value of $1.49 \pm 0.10\, \mu$T and a paleomagnetic dipole moment of $0.25 \pm 0.02 \times 10^{22}$ A m$^{2}$.

The Bushveld paleointensity is strong, comparable to the present-day field and values recorded by SCP analyses from the Archean [11,16], as well as whole-rock paleointensity results from units whose magnetizations appear dominated by single silicates [98,99]. In sharp contrast the Ediacaran Passo da Fabiana value is 30 times weaker, and the lowest time-averaged field strength known to date. Hence, the new data from Huang *et al.* [78] support both the change in field strength leading into the Neoproterozoic and the profound ultra-low fields of the Ediacaran Period (Fig. 6a). The new data from the 591 Ma Passo da Fabiana gabbros together with the 565 Ma Sept Îles paleomagnetic dipole moment [18] suggest an ultra-low time-averaged field interval (UL-TAFI, defined as fields $\le\! 10\%$ of the present day) that lasted at least 26 million years during the Ediacaran Period.

### Further evidence for renewal of the field at 544 Ma

Zhou *et al.* [100] also further examined the timing of the proposed field increase after the UL-TAFI studying the Chatham-Grenville syenite stock of Ontario [101,102]. U-Pb zircon analyses yielded a new age of ca. 544 Ma, some 12 myr older than a previously assigned age based on Ar-Ar analyses of hornblende [103,104]. Zhou *et al.* [100] further applied SCP analysis targeting plagioclase. SEM/EDS analyses indicate that these plagioclase crystals contained titanomagnetite inclusions with SD to pseudosingle sizes, and these domain characteristics were confirmed by magnetic hysteresis and FORC data.

While plagioclase is rare within this syenite, SCP analyses yielded Thellier data passing selection criteria and a field value of $9.9 \pm 2.4\, \mu$T and dipole moment of $2.31 \pm 0.55 \times 10^{22}$ A m$^{2}$. Because the stock likely cooled on a million-year timescale close to the age indicated by U-Pb zircon data, and because of concerns over Ar diffusion and trapped gas in the prior Ar-Ar age data, Zhou *et al.* [100] concluded that ca. 544 Ma provides the best estimate for the magnetization age. The new paleointensity constraint is intermediate between the ultra-low value at 565 Ma reported [18] and the higher value at 532 Ma [77] detailing a relatively rapid increase in field strength consistent with the latest Ediacaran ICN interpretation (Fig. 6a).

There were two other key findings from the Zhou *et al.* [100] study. First Lloyd *et al.* [104] reported a low paleointensity value from a dike interpreted to have been completely reheated by the Chatham-Grenville intrusion. If accurate this value could indicate higher-frequency variations of the field in the earliest Cambrian that might be associated with a very small inner core [105]. However field relations and metamorphic grade indicate that the dike was not heated to temperatures (i.e. $>\! 590^{\circ }$C) sufficient to completely reset any thermoremanent magnetization carried by the dike [100]. Instead the large size of the magnetic grains in the dike suggests that the data reflect an MD ‘pTRM tail’ effect [106,107] and not a true paleofield strength. That is the magnetization of some MD grains may not fully unblock until the Curie temperature ($580^{\circ }$C for magnetite) even if their magnetization was imparted at lower temperature because domain walls are pinned by defects or stress. This ‘pTRM’ tail can greatly underestimate the true field intensity; this effect may have eluded detection because as reported by Lloyd *et al.* [104] the dike they sampled had also been hit by lightning that normally results in exclusion of data because the huge fields can exclude the coercivity of terrestrial magnetic mineral carriers [30]. Nevertheless this provides a cautionary tale revealing that analyses of samples with MD grains common in whole-rock igneous samples can yield low field readings that are incorrect [108,109].

Second successful paleointensity data from syenite plagioclase are significant because this rock type is especially problematic because of the MD grains in bulk samples [110,111]. Syenites can be accurately dated and several occurrences of Neoproterozoic to Cambrian age have the potential to fill crucial gaps in the paleointensity history using SCP analyses. Finally other times for the initiation of inner core growth continue to be discussed and debated (e.g. [112–116]), but none has the combination seen in the Ediacaran ICN age: an ultra-low field defined by robust Thellier time-averaged data and coincident predictions based on thermal evolution models and numerical dynamo simulations [58]. The latter modeling was investigated anew by Davies *et al.* [117] who concluded that the Ediacaran was the most likely time for the initiation of inner core growth.

### Ultra-low-field-aided oxygenation and Ediacaran evolution

**Figure 8.**
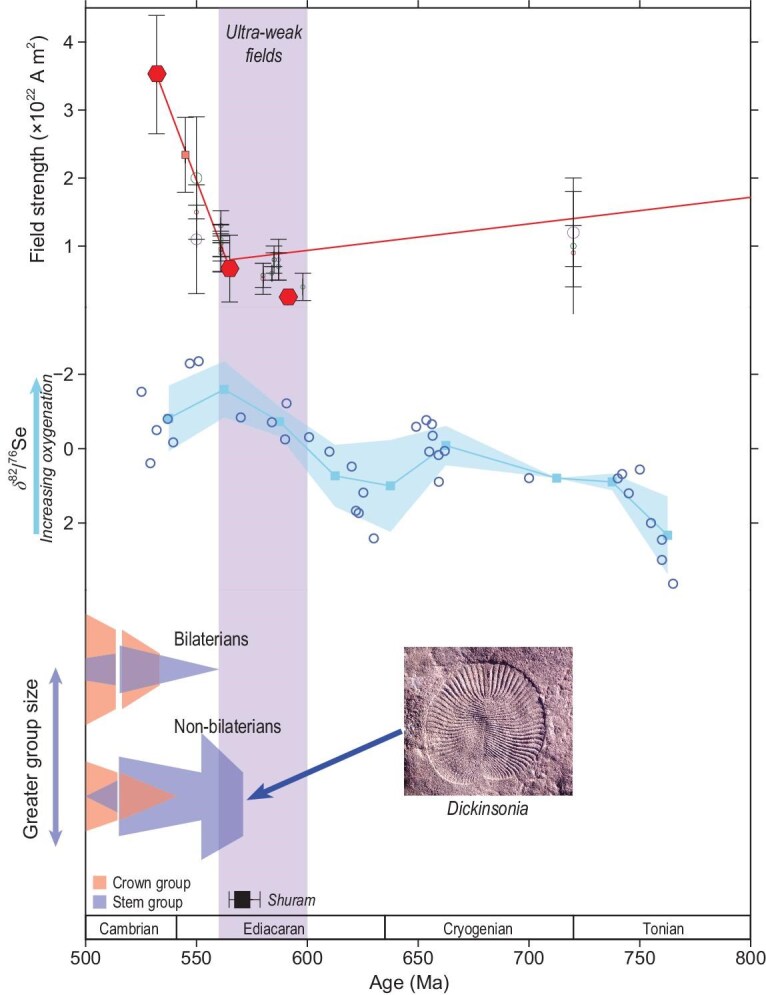
Late Precambrian-Cambrian field strength oxygenation and animal evolution. Top: Tonian to Cambrian field strength (see Fig. 6) with trend lines from Bono *et al.* [18] and Zhou *et al.* [77]. Red symbols are Thellier single-crystal time-averaged data; ultra-low values define an ultra-low time-averaged field interval (UL-TAFI). Also shown are results from whole-rock data from Shcherbakova *et al.* [89], Thallner *et al.* [90,92,93] and Lloyd *et al.* [91] with the following symbols: open circles are results from non-Thellier methods and their sizes are weighted by the number of cooling units; green microwave method; purple Shaw method; black Wilson method; brown open circles Thellier thermal results. Middle: oxygenation from selenium isotopes after Pogge von Strandmann *et al.* [143], sliding window and 1$\sigma$ uncertainty from Huang *et al.* [78]. Bottom: animal evolution after Zhuravlev and Wood [121], Darroch *et al.* [122], Muscente *et al.* [123] and Wood *et al.* [124]. The Shuram isotopic excursion is also shown for reference with ages from Rooney *et al.* [172]. Figure modified from Huang *et al.* [78], licensed under CC BY 4.0.

The idea that evolution of Earth’s core and magnetic field might be related to life has been discussed for over 60 years [1] but only recently has attention focused on the Ediacaran/Cambrian interval. Meert *et al.* [118] and Doglioni *et al.* [119] offered proposals linking Cambrian evolution to a field strength assumed to be low because of the high rate of reversals (i.e. before definition of the ultra-low time-averaged field by Bono *et al.* [18]). They hypothesized increased UV radiation and highlighted its potential to affect DNA but this linkage was questioned [120] because of longstanding arguments [2] for shielding by the atmosphere and ocean. Rather than the Cambrian the UL-TAFI above corresponds with the initial Ediacaran radiation [121–124] of macroscopic animals. That is phylogenomic data suggest that the animal kingdom and many associated phyla diverged before the Ediacaran Period [125–128]. But as documented in the Avalon and White Sea assemblage the diversification of macroscopic animals started at $\sim\! 575$ Ma and reached an apex at $\sim\! 565$ Ma [127–131]. These events show a remarkable correlation with the UL-TAFI (Fig. 8).

The mobile Ediacaran animals reached large sizes (decimeters) $\sim\! 565$ Ma [132–134] and this strongly suggests a higher oxygen demand [135] calling to mind longstanding ideas about the central role oxygenation may have played in the evolution of life [136–139]. There is continued debate about the details of Neoproterozoic oxygenation because of the different sensitivities of various geochemical proxies and evidence for large changes in ocean redox [140–142]. But multiple diverse geochemical approaches yield data suggesting an increase in atmospheric and oceanic O$_{2}$ levels in the late Ediacaran Period specifically in the period of $\sim\! 575$–565 Ma. These include elevated $\delta ^{82/76}$Se values and [Mo] [U] and [V] concentrations in organic-rich shales [143] and pristane/phytane ratios—a redox proxy based on organic geochemistry [144]. Moreover $\delta ^{238}$U on carbonates and $\Delta ^{^{\prime }17}$O-$\delta ^{34}$S-$\delta ^{18}$O of carbonate sulfate suggest ocean oxygenation spanning millions of years during the Shuram excursion [145–147].

Huang *et al.* [78] presented a hypothesis that links the UL-TAFI oxygenation and animal evolution through magnetospheric H loss to space. Using solar wind evolution models [3,11] and the new time-averaged paleointensity data discussed above steady-state solar wind standoff during the UL-TAFI would have been less than 5$r_{\oplus }$ whereas during coronal mass ejection events this distance could compress to 1.6$r_{\oplus }$ intersecting with the plasmasphere [148,149]. This extreme forcing would greatly expand the polar cap area of open field lines where atmospheric escape can occur. Hydrogen would be preferentially lost leaving behind an atmosphere and ocean with increased oxygenation levels. To effect oxygenation, H loss must be substantial, and this requires that supply from the lower atmosphere [150] must have been greater during the UL-TAFI relative to the present. Huang *et al.* [78] noted that with decreased magnetic field greater penetration of highly energetic protons is expected, resulting in the formation of NO$_{{x}}$ compounds that can create ozone holes [151]. With the greater UV flux in a depleted ozone layer more dissociation of water vapor [152] should occur increasing the supply of H available for loss.

As discussed by Huang *et al.* [78] quantifying the H loss is challenging because conditions of the Ediacaran UL-TAFI are so different from those of today. Available models do not include all magnetospheric factors that could lead to enhanced H loss and they lead to a range of predicted loss (e.g. 30% to 10 times following Gunell *et al.* [68] and Egan *et al.* [153] respectively) during the 26 million years of the UL-TAFI. But the greater loss values predict a few percent change of oxygen present atmospheric level (PAL) that might represent the crossing of a threshold aiding the diversification of the large mobile animals of the late Ediacaran.

## SUMMARY DISCUSSION AND OUTLOOK

**Figure 9.**
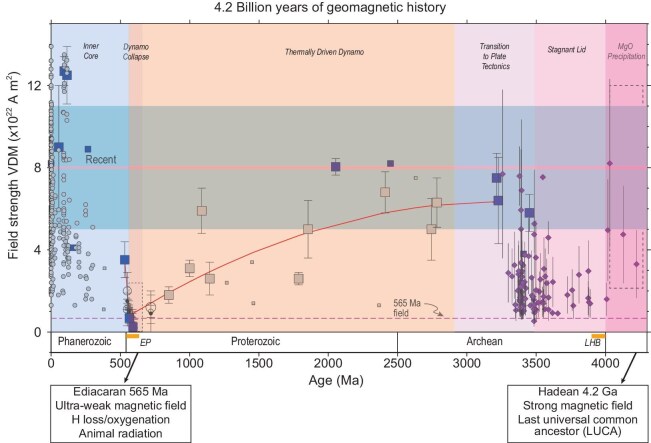
Paleointensity as a tracer of key developments of Earth’s interior, surface and magnetosphere bearing on the origin and evolution of life and habitability. Paleointensity history from extant rocks (see Fig. 6) with constraints from zircon paleomagnetism (Tarduno *et al.* [17,35,36]). The duality of the role of the magnetic field is highlighted. In the Hadean Eon, shielding may have assisted life and emergence of the last universal common ancestor (LUCA) that genetic analyses place at 4.2 Ga (Moody *et al.* [70]) whereas in the Ediacaran Period, the weak field through H escape may have facilitated oxygenation and radiation of large macroscopic animals [78].

Paleointensity data meeting the requirements for robust recording of the mean state of the dynamo remain sparse but substantial progress since the last review extending the history of the geodynamo to 4.2 Ga allows some conclusions to be drawn and working hypotheses to be discussed (Fig. 9). These interpretations are described below, moving from the Hadean and origin of life to the seminal Ediacaran animal radiation.

### Earliest dynamo the origin of life and LUCA

Data indicating a magnetic field at 4.2 Ga [17,35] also provide evidence for a magnetospheric shielding of the atmosphere. This early establishment of the geodynamo is consistent with nitrogen isotopic values indicating limited nitrogen fractionation that would have otherwise been associated with the absence of a global magnetic field [17]. The 4.2 Ga age also corresponds to the time of the LUCA determined by genetic studies [70] (Fig. 9). The presence of a Hadean magnetic field shielding sterilizing solar energetic particles and galactic cosmic rays [120] potentially widens the range of environments for the origin and earliest development of life to include sites without meters of overlying water needed for shielding in the absence of a magnetic field (e.g. shallow lakes [154]). Such life might still need to contend with greater UV radiation [155,156] but estimates of the increase relative to the present depend in turn on model choices for the Hadean atmospheric composition.

LUCA in turn suggests the presence of an early ecosystem and if magnetic shielding was important for this early life an even earlier start to the geodynamo could be expected. Any history of the dynamo prior to 4.2 Ga is in turn linked to events that formed the Moon. The impact of a Mars-sized body (Theia) [157] is the leading hypothesis for the creation of the Moon but there is still uncertainty on the age of the event. For example Halliday and Canup [158] bounded the impact age as 70 to 120 million years after the formation of the Solar System that is established as 4.5673 Ga [159]. On the one hand this impact would have destroyed any pre-existing compositional layering in the core of the proto-Earth that would prohibit convection and a dynamo [160]. On the other hand core superheating associated with the impact might have suppressed a dynamo until that extra heat was lost to the mantle. Recent estimates for the dissipation time are $\sim\! 290$ myr [161]. If core superheating occurred and if this delay time is correct the start of the dynamo might be close in age to the oldest evidence determined from zircon magnetism. If magnetic shielding was important for sustaining early life the time between its origin and LUCA would have been brief on geologic timescales.

Does the potential lack of a dynamo between the lunar-forming event and the appearance of LUCA at 4.2 Ga have significance? In the absence of magnetic shielding the energy supplied by solar energetic particles might have been comparable to that available from the lightning envisioned in the classic experiments of Miller [7] producing amino acids in a highly reducing atmosphere. Sagan [2] hypothesized that in the absence of a magnetic field the influx of solar energetic particles could cause organic molecules to be produced just below the troposphere and that these could be transported to the Earth’s surface through atmospheric convection. More recent magnetohydrodynamic simulations of the intense solar wind conditions in this earliest terrestrial episode—with a geomagnetic field—also predict the formation of organic precursors to life [162] relying in part on funneling of charged particles toward the polar caps. Thus there seem to be dual pathways in which the magnetic field or its absence could have been associated with the formation of the precursors of life. Therefore, further studies defining field presence/absence before 4.2 Ga take on renewed importance.

### Inevitability of water loss and implications for the early Earth

After the high intensity field at ca. 4.1–4.2 Ga, available data record a drop in field intensity and maintenance of these values throughout the Eoarchean era (Fig. 9). The transition to a weaker field and shielding, in light of still intense solar winds [3,11] points toward water loss from the Earth system. This in turn suggests that Earth might have had an early water reservoir greater than that today allowing the present ocean to be a substantial remnant, and/or water was delivered together with the late veneer required to explain siderophile element abundances in the crust [163–165]. Such relatively late water delivery was once thought to be excluded by estimates of the isotopic composition of comets, but more recent data suggest that this assumption may have been premature [166]. A useful comparison is provided by Europa. If the ocean thought to exist on Europa were transferred to Earth by impact it could account for the present terrestrial ocean volume [165]. This long interval of magnetic history and the planetary water budget merits additional study.

### Hadean to Eoarchean stagnant lid stability and low-latitude landmasses

The sustainability of life through the half-billion-year late Hadean to Eoarchean/Paleoarchean interval implies some degree of climatic stability experienced by a given site. One process contrary to that stability is polar wander, whereby the entire Earth might rotate with respect to the spin axis due to changes in its distribution of mass heterogeneities. While more feasible on smaller planetary bodies, the possibility of large polar wander of Earth is highly debated, especially as there are a plethora of magnetic recording pathways [167–169] and inadequacies of frames of reference [170,171] that can lead to false records. But one time interval where we might expect large polar wander is at the onset of plate tectonics, where the penetration of the first slabs into the deep mantle, depending on their global geometry, could change Earth’s moments of inertia. This in turn could cause large and relatively rapid changes in latitudes and associated climatic conditions, that could be deleterious to life, driving its extinction.

The remarkable stability of paleointensity and thus latitude of records from the Jack Hills and Barberton indicate that large true polar wander did not occur throughout the Eoarchean [36]. This is consistent with the conclusion that plate tectonic driving in the form of deep penetration of slabs was not operating derived separately from a comparison of the joint Jack Hills–Barberton record and the history of plate motions for the last 600 million years [36]. These data are consistent with stagnant lid tectonics between 3.9 and ca. 3.4 Ga. and responsible for the crustal generation indicated by the Jack Hills–Barberton zircon record. Plate tectonics is often evoked as being a key factor for the geochemical nutrient cycling important for life. And plate tectonics is likely important for the longer-term heat release from the planet and habitability on the timescale of billions of years. But the evidence that Earth was in a stagnant lid regime in Hadean to early Paleoarchean times indicates that a modern plate tectonic regime is not required for life in the first billion years of the terrestrial planet.

The Jack Hills–Barberton paleolatitude record also indicates that some of the first sites of crustal generation were at low latitudes, and remained there throughout the Eoarchean [36]. These would have provided ideal stable shallow water sites in a potentially equable climate for the development of life. Further research may be able to gain hints as to whether there are more spatial characteristics of sites suitable for Earth’s earliest life.

### Ediacaran field collapse and inner growth

New paleomagnetic data collections as well as a reconsideration of basic parameters of Earth’s core such as thermal conductivity, have led to a quiet revolution in thought about the inner core. Paleointensity data, collected from rocks of three cratonal regions and analyzed by multiple laboratories, record a magnetic field that almost completely collapsed during the Ediacaran Period, being less than 10 times the strength of the present-day field [78] (Fig. 9). This state may have extended over at least 26 million years. The magnetopause standoff distance decreased to $<\! 4.2r_{\oplus }$ and $<\! 1.6r_{\oplus }$ during coronal mass ejections [78]. The magnetic field was restored in the latest Ediacaran/earliest Cambrian times [77] and the attendant return of more robust magnetic shielding probably prevented greater water loss from the planet had the field not been restored. The variable but long-term decrease in field strength prior to the Ediacaran, the ultra-weak state during the period and the rapid increase in strength thereafter makes the latest Ediacaran the prime candidate for the time of nucleation of the solid inner core [77].

### Ediacara fauna and the EMANATE hypothesis

The ultra-weak field corresponds with the radiation of Ediacaran fauna and in particular large mobile forms like *Dickinsonia* that imply greater oxygen demands. Notwithstanding debate, many geochemical proxies show increases of oxygenation at the same time of the faunal radiation and ultra-weak fields. Hence, the question becomes one of what process could link these observations. The linkage proposed was through the feeble Ediacaran magnetosphere that allowed less H from Earth’s atmosphere to be trapped, and the accelerated loss leading to increased oxygenation [78]. Future work is needed to better understand H loss when the geomagnetic field was ultra-weak, for a duration many orders of magnitude longer than our modern observations of the magnetosphere. However, available models predict that primary factors dictating the amount of H loss and hence the associated oxygenation are the duration of the ultra-low field intensity and the strength values throughout the interval. Data are therefore needed for the early Ediacaran and Cryogenian to test the duration, whereas more Ediacaran data are needed to explore whether the field was persistently low, and how weak the field became. Studies of whether environmental conditions in the Ediacaran, including those during the Shuram isotopic excursion [172] (Fig. 8), facilitated H delivery to the upper atmosphere, are also needed.

The radiation of macroscopic and mobile animals in the Ediacaran Period was a striking step forward in evolution. Debate continues on the relationship between the Ediacara fauna and modern forms, but thoughts are arguably changing. Rather than being viewed as a separate experiment in evolution, the Ediacara fauna may be the earliest start of a continuum leading into the Cambrian explosion of life [173], and therefore there may be genetic ties between these early forms and subsequent animals, ultimately leading to intelligent life. This potential linkage highlights the importance of understanding the role of the extremely weak magnetic field. If correct, our proposition, called here the EMANATE hypothesis for short (Earth’s magnetism allowed new animals to evolve), represents a new twist in how Earth’s magnetic field is related to life. In this case, it was the weakness of the field, rather than its strong intensity and standoff of the solar wind, that was the key factor that assisted this seminal event in animal evolution.

## Data Availability

Data presented here have been published previously and are available in the respective citations. Figure panels used are licensed under CC BY 4.0 and CC BY-NC-ND 4.0.
